# Global Epidemiology of Bat Coronaviruses

**DOI:** 10.3390/v11020174

**Published:** 2019-02-20

**Authors:** Antonio C. P. Wong, Xin Li, Susanna K. P. Lau, Patrick C. Y. Woo

**Affiliations:** 1 Department of Microbiology, Li Ka Shing Faculty of Medicine, The University of Hong Kong, Pokfulam, Hong Kong; antonwcp@hku.hk (A.C.P.W); lixinlyh@connect.hku.hk (X.L.); 2 State Key Laboratory of Emerging Infectious Diseases, The University of Hong Kong, Pokfulam, Hong Kong; 3 Research Centre of Infection and Immunology, The University of Hong Kong, Pokfulam, Hong Kong; 4 Carol Yu Centre for Infection, The University of Hong Kong, Pokfulam, Hong Kong; 5 Collaborative Innovation Centre for Diagnosis and Treatment of Infectious Diseases, The University of Hong Kong, Pokfulam, Hong Kong

**Keywords:** global, epidemiology, bat, coronavirus, *Alphacoronavirus*, *Betacoronavirus*, interspecies transmission, host

## Abstract

Bats are a unique group of mammals of the order *Chiroptera*. They are highly diversified and are the group of mammals with the second largest number of species. Such highly diversified cell types and receptors facilitate them to be potential hosts of a large variety of viruses. Bats are the only group of mammals capable of sustained flight, which enables them to disseminate the viruses they harbor and enhance the chance of interspecies transmission. This article aims at reviewing the various aspects of the global epidemiology of bat coronaviruses (CoVs). Before the SARS epidemic, bats were not known to be hosts for CoVs. In the last 15 years, bats have been found to be hosts of >30 CoVs with complete genomes sequenced, and many more if those without genome sequences are included. Among the four CoV genera, only alphaCoVs and betaCoVs have been found in bats. As a whole, both alphaCoVs and betaCoVs have been detected from bats in Asia, Europe, Africa, North and South America and Australasia; but alphaCoVs seem to be more widespread than betaCoVs, and their detection rate is also higher. For betaCoVs, only those from subgenera *Sarbecovirus*, *Merbecovirus*, *Nobecovirus* and *Hibecovirus* have been detected in bats. Most notably, horseshoe bats are the reservoir of SARS-CoV, and several betaCoVs from subgenus *Merbecovirus* are closely related to MERS-CoV. In addition to the interactions among various bat species themselves, bat–animal and bat–human interactions, such as the presence of live bats in wildlife wet markets and restaurants in Southern China, are important for interspecies transmission of CoVs and may lead to devastating global outbreaks.

## 1. Introduction

**Figure 1:**
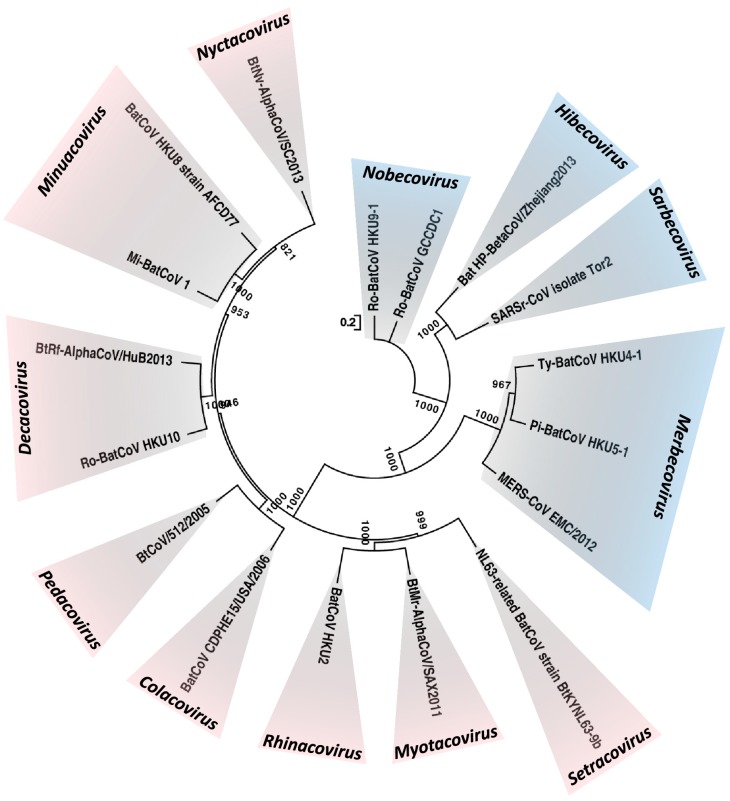
Maximum-likelihood phylogeny based on the complete genome sequences of 17 bat CoV species released by ICTV in 2018. A general time-reversible model of nucleotide substitution with estimated base frequencies, the proportion of invariant sites, and the γ distribution of rates across sites were used in the maximum-likelihood analysis. Bootstrap values are shown next to the branches. The scale bar indicates the number of nucleotide substitutions per site. Different colors represent different genera. Red, *Alphacoronavirus*; blue, *Betacoronavirus*. Updated subgenera clusters are labelled *Setracovirus, Myotacovirus, Rhinacovirus, Colacovirus, Pedacovirus, Decacovirus, Minunacovirus, Nyctacovirus* for the *Alphacoronavirus* and *Nobecovirus, Hibecovirus, Sarbecovirus, Merbecovirus* for the *Betacoronavirus*.

Coronaviruses (CoVs) infect humans and a wide variety of animals, causing diseases in the respiratory, enteric, hepatic, and neurological systems with varying severity. CoVs are classified into four genera, *Alphacoronavirus*, *Betacoronavirus*, *Gammacoronavirus* and *Deltacoronavirus*. Within *Betacoronavirus*, they can be further subclassified into lineages A, B, C and D [1]. In 2018, these four lineages were reclassified as subgenera of *Betacoronavirus*, and renamed as *Embecovirus* (previous lineage A), *Sarbecovirus* (previous lineage B), *Merbecovirus* (previous lineage C) and *Nobecovirus* (previous lineage D) [2]. In addition, a fifth subgenus, *Hibecovirus*, was also included (Figure 1) [2]. As a result, of the unique mechanism of viral replication, CoVs have a high frequency of recombination [3,4,5,6,7,8]. CoVs may achieve rapid adaptation to new host and ecological niches, as a result of their tendency for recombination and the inherently high mutation rates, which are typical of RNA viruses [9]. 

Bats are a unique group of mammals of the order *Chiroptera*. Traditionally, bats have been classified into two suborders: the megabats, which are mostly frugivorous, and the microbats, which are mostly insectivorous. However, recent molecular studies have supported a revision of the classification into the suborders *Yinpterochiroptera* and *Yangochiroptera*, of which *Yinpterochiroptera* consists of the megabats and several microbat species [10]. Bats are globally distributed, although individual bat species have their own geographical niche [11]. Bats are highly diversified and are the group of mammals with the second largest number of species [12]. Such high diversification of bat species provides various cell types and receptors which facilitate them being potential hosts to a large variety of viruses. Bats are the only group of mammals capable of sustained flight, which enables them to disseminate the viruses they harbor and enhance the chance of interspecies transmission. Traditionally, bats are recognized to be hosts of several highly pathogenic viruses, such as rabies virus and other lyssaviruses, Hendra virus, Nipah virus and Ebola virus [13,14,15,16,17,18,19,20,21]. 

Before the Severe Acute Respiratory Syndrome (SARS) epidemic, bats were not known to be hosts for CoVs. After the SARS epidemic, there was a boost in interest regarding searching for novel CoVs in various mammals. In the last 14 years, bats have been found to be hosts of at least 30 CoVs with complete genome sequences available [7,22,23,24,25,26,27,28,29,30,31,32], and many more if those without genome sequences are included [33,34,35,36,37,38,39,40,41,42]. Most notably, horseshoe bats were found to be the reservoir of SARS-like CoVs, while palm civet cats are considered to be the intermediate host for SARS-CoVs [43,44,45]. In addition, several betaCoVs from subgenus *Merbecovirus* were also found to be closely related to the Middle East Respiratory Syndrome (MERS)-CoV in dromedary camels and humans [23,29,46,47]. In this article, we review the various aspects of the global epidemiology of bat CoVs. The detailed molecular evolution, phylogenetic analysis and recombination studies, which have been reviewed by others [48,49,50], will not be included in this review.

## 2. What CoVs are Found in Bats?

Among the four CoV genera, only alphaCoVs and betaCoVs have been found in bats. In fact, this is the basis of our theory that bat CoVs are the ancestors for alphaCoVs and betaCoVs, whereas bird CoVs are the ancestors for gammaCoVs and deltaCoVs [51]. Interestingly, for the betaCoVs, only those from subgenera *Sarbecovirus* (SARS-related CoVs)*, Merbecovirus* (Ty-BatCoV HKU4, Pi-BatCoV HKU5, Hp-BatCoV HKU25, MERS-related CoVs)*, Nobecovirus (*Ro-BatCoV HKU9 and Ro-BatCoV GCCDC1) and *Hibecovirus* (Bat Hp-betaCoV Zhejiang2013) have been detected in bats so far [2,23,24,27,28,29,52]. As several betaCoVs from the subgenus *Embecovirus* (Murine CoV and ChRCoV HKU24) have been discovered in rodents, and this group of mammals is the one with the second largest number of species, we speculate that rodent CoVs may be the ancestors of *Embecovirus* [2,53].

## 3. Bat-Animal and Bat-Human Interactions: Interspecies Jumping of Bat CoVs

In addition to the interactions among the various bat species themselves, interactions between bats and other animals, as well as interactions between bats and human are important for interspecies transmission of viruses. Scientists have proposed several possible activities or events that have led to successful interspecies jumping of CoVs in the last decade. For instance, bats are used as food in Southern China and other countries in Southeast Asia. Live bats are found in wild life wet markets and restaurants in Southern China, which have greatly facilitated bat-human and bat-animal interactions. One of the first cases of SARS occurred in a chef from Heyuan. He worked in a restaurant in Shenzhen and had regular contact with wild food animals, including bats [54]. Shortly afterwards, SARS-CoVs were isolated from caged Himalayan palm civets from wild live markets in Guangdong [55,56,57]. There are many different predators of bats, and what could consume them depends on their locations. Some flying animals, such as owls and hawks, are natural predators of bats. Owls can be active at night when the bats are out. Owls are able to capture bats without any warning while they are in flight [58]. Weasels and raccoons have also been identified as bat predators in some locations and they often lurk around places where the bats reside; and interestingly, SARS-CoV was also detected in a raccoon dog in a Chinese wet market [57,59]. All these interactions between bats and other animals and between bats and human may contribute to CoV interspecies jumping. 

Theoretically, in order to achieve successful interspecies jumping, several conditions need to be satisfied. Firstly, a host reservoir of CoVs should be established. Numerous surveillance studies conducted by different groups of scientists have proven that bats serve as a major reservoir of alphaCoVs and betaCoVs [7,20,22,25,42,51,52,60,61,62,63,64,65]. The wide diversity of bat species and their capability of sustained flight allow them to occupy a broad worldwide habitat. 

Secondly, direct or indirect transmission routes between donor and recipient hosts should be identified. However, there are few reports on direct contact between bats and human or other animals, except for consumption by bat predators or human [58]. Yet, the possibility of spillover events should not be overlooked. It is believed that the spillover of bat CoVs mainly occurs by host viral shedding, an indirect route whereby interspecies transmission could be achieved. 

Thirdly, when viral spillover events are considered, tissue tropism of CoVs is a major determining factor [66]. Unlike Hendra virus and Nipah virus, both alphaCoVs and betaCoVs have much higher detection rates in intestinal and fecal samples than in throat or urine samples, although the donor host may not manifest apparent pathologies or diseases [7,43,67,68,69]. Therefore, bat excretions are the major environmental source shedding CoVs in spillover events. The close contact between the recipient host’s susceptible tissue and bat excretions or contaminated fomites becomes the essential link for transmission. 

Fourthly, survival fitness of CoVs in the environment determines the chance of successful spillover events. CoVs are enveloped, positive-sense single-stranded RNA viruses with surface spike protein projection. A high genomic mutation rate allows CoVs to evolve, and leads to high diversity with potential for host receptor adaptation [4,5,7,70]. However, the presence of viral lipid envelopes renders CoVs sensitive to environmental conditions such as desiccation, heat, extreme pH, UV light and presence of detergents [71]. Prolonged exposure to unfavorable natural environments leads to rapid viral decay and loss of infectivity [71,72,73]. Therefore, the particular location of viral shedding events and relevant environmental condition should be taken into account when predicting the potential of interspecies transmission. 

Finally, to ultimately complete the spillover chain, susceptible recipient hosts need to be present within the viral shedding area. Host susceptibility mainly depends on the availability of specific receptors on recipient tissue to interact with the spike protein of CoVs for viral entry. The receptor profile of several CoV species has been well studied, for example angiotensin-converting enzyme 2 (ACE2) for SARS-CoV and HCoV NL63, dipeptidyl peptidase-4 (DPP4) for MERS-CoV, aminopeptidase-N (APN) for HCoV 229E, TGEV and PRCV, carcinoembryonic antigen-related cell adhesion molecule 1 (CECAM1) for MHV and sugar receptor for BCoV [59,74,75,76,77,78,79,80,81,82,83,84,85,86,87,88,89,90,91,92]. However, the bat CoV receptor profile is relatively understudied. A few proteomic studies on several CoV species from *Merbecovirus* and *Sarbecovirus* revealed possible usage of DPP4 and ACE2 receptors respectively, but in a gradient of binding affinity towards different hosts’ receptors [24,74,93,94,95]. Yet, except for a few strains of SARS-like CoV, most of these bat CoVs were never reported to be successfully isolated or pathogenically studied [22,23,24,25,26,27,29,43,61,65]. It remains unknown whether the majority of these bat CoVs have pathogenic potential in humans. 

This notwithstanding, available evidence provides insight that recipient hosts which share similar receptor identity to human are potential candidates as intermediate hosts in interspecies jumping events [96,97,98,99]. A common phenomenon observed in both SARS-CoV and SADS-CoV outbreaks is that the outbreaks involved caged or farmed animals which were restricted in defined areas with bats residing around [55,56,63,100,101,102]. In the case of SARS-CoV, civets in the wild were found to be free from the infection [43]. In other words, human activities facilitated the viral spillover events by bringing susceptible recipient hosts to the vicinity of viral sources. Surveillance on interspecies transmission should be placed around wet markets, farms and abattoirs to safeguard human from novel zoonotic diseases. 

## 4. Geographical Distribution of Bat CoVs

**Figure 2:**
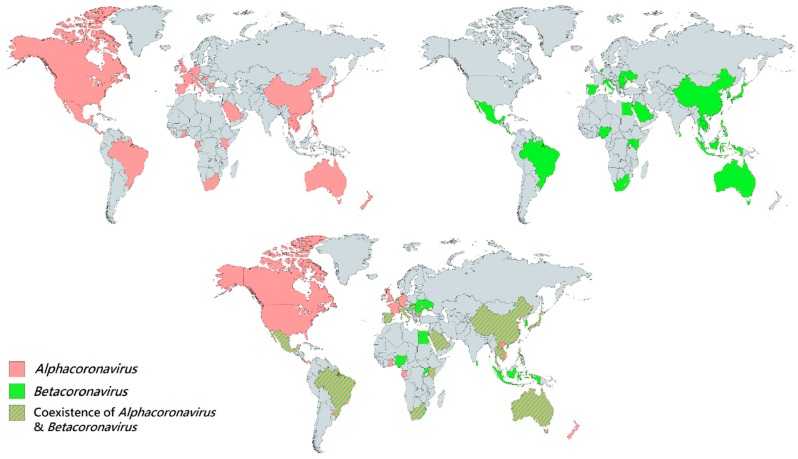
Geographical distribution of bat CoVs from the genera *Alphacoronavirus* and *Betacoronavirus*. Each colored region represents the country which reported the discovery of bat CoV. Red regions represent the countries which discovered bat *Alphacoornavirus*. Green regions represent the countries which discovered bat *Betacoronavirus*. Red-green striped regions represent the countries which discovered both bat *Alphacoronavirus* and *Betacoronavirus*.

**Figure 3:**
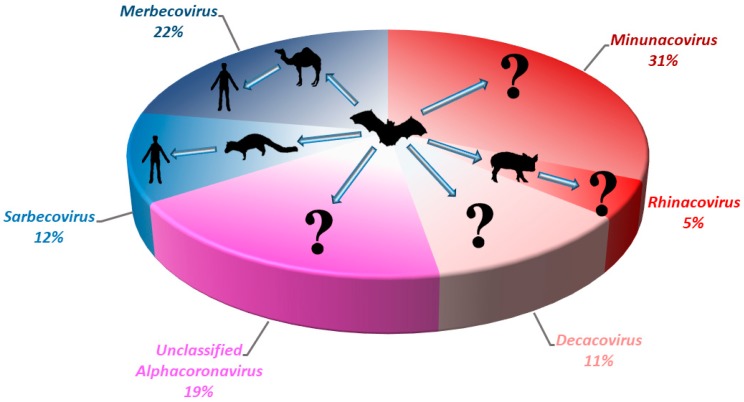
Pie chart showing the relative detection rate of different bat CoVs from different subgenera of *Alphacoronavirus* and *Betacoronavirus* in Hong Kong from 2008 to 2017. The potential zoonotic transmission routes of each sub-genus of bat CoV detected are shown. Unclassified *Alphacoronavirus* represents those without complete genome sequences or genome characterization. Red color represents the sub-genera from *Alphacoronavirus*; Blue color represents the sub-genera from *Betacoronavirus*.

**Figure 4:**
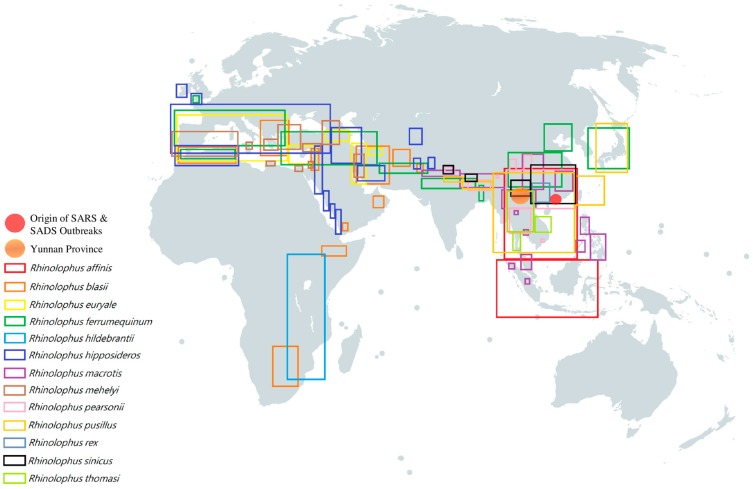
Geographical distribution of different horseshoe bats which were discovered to carry SARS-like BatCoV [114,115,116,117,118,119,120,121,122,123,124,125]. Each colored rectangular box represents the geographical distribution of a specific horseshoe bat species respectively: red box, *Rhinolophus affinis*; orange box, *Rhinolophus blasii*; yellow box, *Rhinolophus euryale*; green box, *Rhinolophus ferrumequinum*; turquoise box, *Rhinolophus hildebrantii*; indigo box, *Rhinolophus hipposideros*; purple box, *Rhinolophus macrotis*; brown box, *Rhinolophus mehelyi*; pink box, *Rhinolophus pearsonii*; gold box, *Rhinolophus pusillus*; blue-gray box, *Rhinolophus rex*; black box, *Rhinolophus sinicus*; lime box, *Rhinolophus thomasi*. Orange circle represents Yunnan Province; Red circle represents the origin of SARS & SADS outbreaks.

Generally, both alphaCoVs and betaCoVs have been detected in bats in Asia, Europe, Africa, North and South America and Australasia [7,22,30,37,42,65,68,103,104,105,106,107,108,109,110]. In general, alphaCoVs seem to be more widespread than betaCoVs (Figure 2), and their detection rate is also higher. In our experience, the prevalence of alphaCoVs was around twice that of betaCoVs for bats in Hong Kong (Figure 3) (unpublished data). Regional patterns of bat CoV outbreaks at species level can be deduced from the population distribution of their respective bat hosts. There were three major CoV outbreaks in the past 15 years, including SARS-CoV from *Sarbecovirus*, MERS-CoV from *Merbecovirus* and SADS-CoV from *Rhinacovirus* [54,63,100,101,111,112]. Apart from MERS-CoV, whose most recent common ancestor still remains unknown, bats have been confirmed to be the origin of SARS-CoV and SADS-CoV [7,43,100,101,113]. Interestingly, both SARS-CoV and SADS-CoV were discovered in horseshoe bats, mainly *Rhinolophus sinicus* and *Rhinolophus affinis* [7,100]. The outbreaks were located in southeast China, where a large diversity of horseshoe bats resides, especially the two species (*Rhinolophus sinicus* and *Rhinolophus affinis*), mentioned above (Figure 4) [7,100]. This suggests that the next CoV outbreak can be geographically predicted by the specific bat species’ distribution.

**Figure 5:**
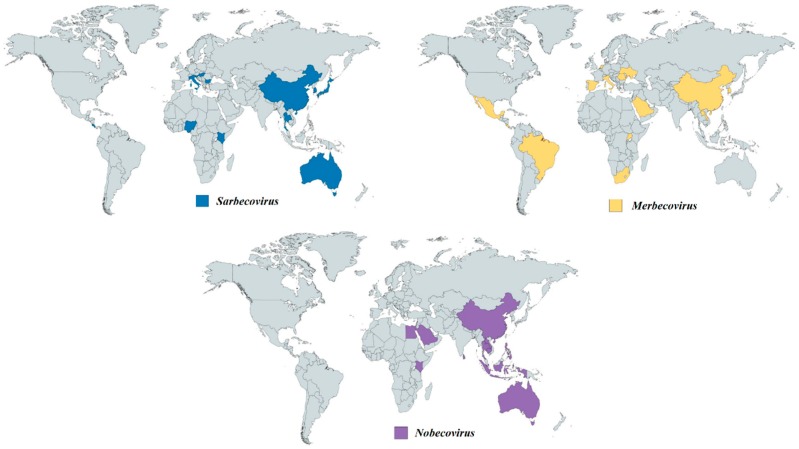
Geographical distribution of bat CoVs from the genus *Betacoronavirus*. Each colored region represents the country which reported the discovery of bat CoV from different sub-genera. Navy-blue regions represent the countries which discovered bat CoVs from *Sarbecovirus*. Yellow regions represent the countries which discovered bat CoVs from *Merbecovirus*. Purple regions represent the countries which discovered bat CoVs from *Nobecovirus*.

Several hypotheses have been proposed based on available evidence regarding the contributions of bat species diversity together with their respective habits towards CoV epidemiology. Firstly, since both SARS-like CoV and SADS-like CoV are found in both Indomalaya and Palearctic biogeographic realms and a few horseshoe bat species like *Rhinolophus ferrumequinum* reside across both realms, these horseshoe bats may act as a bridge to carry the CoVs from one realm to another (Figure 4) [44,62,122,126,127,128,129,130,131]. Secondly, two CoV species from *Nobecovirus,* Ro-BatCoV HKU9 and Ro-BatCoV GCCDC1, are found in *Rousettus leschenaultia*, a species of fruit bats located in southern Asia [27,28,60]. Later studies also discovered Ro-BatCoV HKU9 from other species of *Rousettus* bats and Ro-BatCoV GCCDC1 from *Eonycteris spelaea* [132]. Most of the *Rousettus* and *Eonycteris* bats reside within the Indomalaya realm, with a few species of *Rousettus* bats found in the Afrotropic realm (Figure 5). Therefore, these ecozones may become hotspots for potential interspecies jumping events of *Nobecovirus.* Thirdly, *Merbecovirus* diversity is positively correlated with the diversity of bats in the family *Vespertilionidae.* It is observed that CoVs from *Merbecovirus* are able to infect different bat genera in the large *Vespertilionidae* family with distinct and diverse habitats, providing a clue that *Merbecovirus* is more widespread geographically than *Sarbecovirus* and *Norbecovirus* (Figure 5) [23,24,28,29,32,61].

### 4.1. Sarbecovirus (betaCoVs): SARS-like CoV in Horseshoe Bats

Since the first discovery of SARS-like CoV in Chinese horseshoe bats in Hong Kong in 2005, several molecular epidemiology studies have been carried out to look for this highly fatal CoV globally [7,43,44,64,126,127,129,130,133]. Several conclusions can be drawn from these studies. Firstly, most bat SARS-like CoVs were detected in various species of horseshoe bats (genus *Rhinolophus* of suborder *Yinpterochiroptera*), although a few strains have also been found in *Aselliscus stoliczkanus*, *Chaerephon plicatus* and *Hipposideros larvatus* [64,109,134]. So far, the largest population of completely sequenced bat SARS-like CoVs were found in Chinese horseshoe bats (*Rhinolophus sinicus*) [7,64]. However, there are not sufficient data to establish the prevalence of SARS-like CoVs in different bat host species, especially the species under the genus *Rhinolophus*. Interestingly, geographical factor does contribute to the diversity of SARS-like CoVs. Available genome sequences showed that the majority of SARS-like CoVs found in Yunnan province shared higher nucleotide identity with human and civet SARS-CoVs in the range of 90–95%, while those found in southeast China, Korea and Europe shared only 77–90% genome nucleotide identity (unpublished data) [64,129,131,135]. However, despite having significantly different genomes, especially in the receptor binding domain where over 20% nucleotide differences were observed, both the SARS-like CoVs found in Yunnan province (southwest China) and those from southeast China were able to infect Chinese horseshoe bats (unpublished data). Further knowledge of the interaction between SARS-like CoVs and bat host receptors will shed light upon this issue. Moreover, bat SARS-like CoVs are only found in Asia, Europe and Africa, but not America or Australasia (Figure 5). This is probably because of a relatively small population of horseshoe bats in America and Australasia. Moreover, Yunnan Province in China is an ecological niche where many different species of horseshoe bats reside (Figure 4). It is also the geographical region where the highest diversity of bat SARS-like CoV was observed, with many recombination events among the various strains of bat SARS-like CoV [64,135,136]. In fact, Yunnan is one of the provinces with the highest animal diversity in China, therefore harboring a large number of viruses and facilitating their recombination and interspecies jumping events [137]. Intriguingly, there are no observable pathogenic features reported in horseshoe bats that are infected with SARS-like CoVs [7,138]. In fact, there are few reports on bats being symptomatic due to either alphaCoVs or betaCoVs infection. This special feature makes bats the ideal reservoir hosts for CoVs.

### 4.2. Merbecovirus (betaCoVs)

When MERS-CoV was first isolated in the Middle East in 2012 and its genome sequenced; it was found that MERS-CoV was most closely related to Ty-BatCoV HKU4 in *Tylonycteris pachypus* and Pi-BatCoV HKU5 in *Pipistrellus abramus*, which were the only known members of *Merbecovirus* at that time [28,46]. Since the emergence of MERS-CoV, more than 10 additional betaCoVs of the subgenus *Merbecovirus* have been discovered from various bats of suborder *Yangochiroptera* in Asia, Europe, Africa and North and South America, most commonly from bats of family *Vespertilionidae* [23,24,29,31,32,35,42,61,105,110]. So far, no betaCoVs from the sub-genus *Merbecovirus* have been detected in bats of suborder *Yinpterochiroptera*. However, unlike SARS-CoV, of which most of the genome sequences of bat SARS-CoVs are >90% identical to those of civet and human SARS-CoVs, the genome sequences of MERS-CoV in human and dromedaries possess only around 65–80% nucleotide identities to those of the other members of subgenus *Merbecovirus* from different bats [23,24,29,31,32,46,61]. Compared to *Sarbecovirus* and *Nobecovirus*, the bat hosts that harbor viruses from the *Merbecovirus* sub-genus are more diverse. This is in line with the presence of more CoV species belonging to *Merbecovirus*. 

### 4.3. Nobecovirus (betaCoVs)

Ro-BatCoV HKU9 and Ro-BatCoV GCCDC1 are the two CoV species from the sub-genus *Nobecovirus*. Compared with other bat CoVs from *Sarbecovirus* and *Merbecovirus*, *Nobecovirus* involves fewer bat host species. This might be due to their host specificity towards fruit bats like *Rousettus* bats and *Eonycteris* bats, which belong to the family *Pteropodidae* [28,60,132]. The majority of bat species from the *Pteropodidae* family are found in tropical and subtropical areas, which is consistent with the locations at which Ro-BatCoV HKU9 and Ro-BatCoV GCCDC1 are reported [27,60,65,132]. Several other deadly zoonotic viruses are also found in several bats belonging to the *Pteropodidae* family by molecular detection or viral isolation, including Ebola virus from *Epomops franqueti*, *Hypsignathus monstrosus* and *Myonycteris torquata*, Marburg virus from *Rousettus aegyptiacus*, Hendra virus from *Pteropus alecto*, *Pteropus conscpicillatus*, *Pteropus poliocephalus* and *Pteropus scapulatus*, and Nipah virus from *Pteropus lylei* and *Pteropus hypomelanus* [14,139,140,141,142,143]. Pteropine orthoreovirus (PRV) is a zoonotic virus from the family *Rheoviridae* discovered from humans and *Pteropus* bats [144]. Interestingly, Ro-BatCoV GCCDC1 has been reported to harbor a p10 fusogenic gene, which was obtained from *orthoreovirus* [27]. This evidence suggests that CoVs are capable of undergoing recombination with viruses from other families, especially in the case of *Nobecovirus,* which share similar host tropism with the deadly viruses like Ebola virus, Marburg virus, Hendra virus and Nipah viruses. The zoonotic potential of *Nobecovirus* should not be underestimated, and surveillance of the *Pteropodidae* family in tropical and subtropical regions like Southeast Asia for emerging CoVs is necessary for future outbreak precaution. 

## 5. Concluding Remarks

In the last 15 years, we have witnessed a large number of novel CoVs being discovered. Surprisingly, bats are the group of mammals that harbor the largest number of CoVs. All these discoveries and the genomes sequenced have given us unprecedented opportunities to understand the evolution of CoVs as well as the paths of interspecies transmission, which sometimes have led to devastating outbreaks, such as SARS and MERS. In the next decade, using the genome information and with the help of sophisticated technologies such as construction of wild-type and mutant infectious clones, we would be able to design experiments to further dissect the molecular mechanism of interspecies jumping. Moreover, more intensive surveillance should be performed in geographical areas which are relatively under-studied, so that a more comprehensive and detailed picture of the global epidemiology can be grasped.

## Abbreviations

ACE2	Angiotensin-converting enzyme 2
AlphaCoV	Alphacoronavirus
APN	Aminopeptidase-N
BCoV	Bovine coronavirus
BetaCoV	Betacoronavirus
CECAM1	Carcinoembryonic antigen-related cell adhesion molecule 1
ChR	China Rattus
CoVs	Coronaviruses
DeltaCoV	Deltacoronavirus
DPP4	Dipeptidyl peptidase-4
GammaCoV	Gammacoronavirus
HCoV	Human coronavirus
Hp	Hypsugo pulveratus
MERS-CoV	Middle East Respiratory Syndrome coronavirus
MHV	Murine hepatitis virus
Pi	Pipistrellus
PRCV	Porcine respiratory coronavirus
Ro	Rousettus
SADS-CoV	Swine Acute Diarrhea Syndrome coronavirus
SARS-CoV	Severe Acute Respiratory Syndrome coronavirus
TGEV	Transmissible gastroenteritis coronavirus
Ty	Tylonycteris
UV	Ultraviolet

## References

[1] De Groot R.J., Baker S.C., Baric R., Enjuanes L., Gorbalenya A.E., Holmes K.V., Perlman S., Poon L., Rottier P.J.M., Talbot P.J., King A.M.Q., Adams M.J., Carstens E.B., Lefkowitz E.J. (2011). Family Coronaviridae. Virus Taxonomy, Classification and Nomenclature of Viruses. Ninth Report of the International Committee on Taxonomy of Viruses.

[2] ICTV Taxonomy History: Cornidovirineae. https://talk.ictvonline.org/taxonomy/p/taxonomy-history?taxnode_id=20186105.

[3] Lau S.K., Woo P.C., Yip C.C., Tse H., Tsoi H.W., Cheng V.C., Lee P., Tang B.S., Cheung C.H., Lee R.A. (2006). Coronavirus HKU1 and other coronavirus infections in Hong Kong. J. Clin. Microbiol..

[4] Graham R.L., Baric R.S. (2010). Recombination, reservoirs, and the modular spike: Mechanisms of coronavirus cross-species transmission. J. Virol..

[5] Lai M.M., Baric R.S., Makino S., Keck J.G., Egbert J., Leibowitz J.L., Stohlman S.A. (1985). Recombination between nonsegmented RNA genomes of murine coronaviruses. J. Virol..

[6] Tian P.F., Jin Y.L., Xing G., Qv L.L., Huang Y.W., Zhou J.Y. (2014). Evidence of recombinant strains of porcine epidemic diarrhea virus, United States, 2013. Emerg. Infect. Dis..

[7] Lau S.K.P., Li K.S.M., Huang Y., Shek C.T., Tse H., Wang M., Choi G.K.Y., Xu H., Lam C.S.F., Guo R. (2010). Ecoepidemiology and Complete Genome Comparison of Different Strains of Severe Acute Respiratory Syndrome-Related Rhinolophus Bat Coronavirus in China Reveal Bats as a Reservoir for Acute, Self-Limiting Infection That Allows Recombination Events. J. Virol..

[8] Decaro N., Mari V., Campolo M., Lorusso A., Camero M., Elia G., Martella V., Cordioli P., Enjuanes L., Buonavoglia C. (2009). Recombinant canine coronaviruses related to transmissible gastroenteritis virus of Swine are circulating in dogs. J. Virol..

[9] Holmes E.C., Rambaut A. (2004). Viral evolution and the emergence of SARS coronavirus. Philos. Trans. R. Soc. Lond. B. Biol. Sci..

[10] Tsagkogeorga G., Parker J., Stupka E., Cotton J.A., Rossiter S.J. (2013). Phylogenomic analyses elucidate the evolutionary relationships of bats. Curr. Biol..

[11] Bats of the World. https://cdn.bats.org.uk/pdf/Bats-of-the-World.pdf?mtime=20181101151316.

[12] Jones K.E., MacLarnon A. (2001). Bat life histories: testing models of mammalian life-history evolution. Evol. Ecol. Res..

[13] Chua K.B., Bellini W.J., Rota P.A., Harcourt B.H., Tamin A., Lam S.K., Ksiazek T.G., Rollin P.E., Zaki S.R., Shieh W. (2000). Nipah virus: A recently emergent deadly paramyxovirus. Science.

[14] Leroy E.M., Kumulungui B., Pourrut X., Rouquet P., Hassanin A., Yaba P., Delicat A., Paweska J.T., Gonzalez J.P., Swanepoel R. (2005). Fruit bats as reservoirs of Ebola virus. Nature.

[15] Halpin K., Young P.L., Field H.E., Mackenzie J.S. (2000). Isolation of Hendra virus from pteropid bats: A natural reservoir of Hendra virus. J. Gen. Virol..

[16] Badrane H., Tordo N. (2001). Host switching in Lyssavirus history from the Chiroptera to the Carnivora orders. J. Virol..

[17] Suu-Ire R., Begeman L., Banyard A.C., Breed A.C., Drosten C., Eggerbauer E., Freuling C.M., Gibson L., Goharriz H., Horton D.L. (2018). Pathogenesis of bat rabies in a natural reservoir: Comparative susceptibility of the straw-colored fruit bat (Eidolon helvum) to three strains of Lagos bat virus. PLoS Negl. Trop. Dis..

[18] Streicker D.G., Winternitz J.C., Satterfield D.A., Condori-Condori R.E., Broos A., Tello C., Recuenco S., Velasco-Villa A., Altizer S., Valderrama W. (2016). Host-pathogen evolutionary signatures reveal dynamics and future invasions of vampire bat rabies. Proc. Natl. Acad. Sci. USA.

[19] Francis J.R., McCall B.J., Hutchinson P., Powell J., Vaska V.L., Nourse C. (2014). Australian bat lyssavirus: implications for public health. Med. J. Aust..

[20] Warrilow D., Harrower B., Smith I.L., Field H., Taylor R., Walker C., Smith G.A. (2003). Public health surveillance for Australian bat lyssavirus in Queensland, Australia, 2000–2001. Emerg. Infect. Dis..

[21] Guyatt K.J., Twin J., Davis P., Holmes E.C., Smith G.A., Smith I.L., Mackenzie J.S., Young P.L. (2003). A molecular epidemiological study of Australian bat lyssavirus. J. Gen. Virol..

[22] Lau S.K., Woo P.C., Li K.S., Huang Y., Wang M., Lam C.S., Xu H., Guo R., Chan K.H., Zheng B.J. (2007). Complete genome sequence of bat coronavirus HKU2 from Chinese horseshoe bats revealed a much smaller spike gene with a different evolutionary lineage from the rest of the genome. Virology.

[23] Corman V.M., Ithete N.L., Richards L.R., Schoeman M.C., Preiser W., Drosten C., Drexler J.F. (2014). Rooting the Phylogenetic Tree of Middle East Respiratory Syndrome Coronavirus by Characterization of a Conspecific Virus from an African Bat. J. Virol..

[24] Lau S.K.P., Zhang L., Luk H.K.H., Xiong L., Peng X., Li K.S.M., He X., Zhao P.S., Fan R.Y.Y., Wong A.C.P. (2018). Receptor Usage of a Novel Bat Lineage C Betacoronavirus Reveals Evolution of Middle East Respiratory Syndrome-Related Coronavirus Spike Proteins for Human Dipeptidyl Peptidase 4 Binding. J. Infect. Dis..

[25] Lau S.K., Li K.S., Tsang A.K., Shek C.T., Wang M., Choi G.K., Guo R., Wong B.H., Poon R.W., Lam C.S. (2012). Recent transmission of a novel alphacoronavirus, bat coronavirus HKU10, from Leschenault’s rousettes to pomona leaf-nosed bats: First evidence of interspecies transmission of coronavirus between bats of different suborders. J. Virol..

[26] Woo P.C., Lau S.K., Li K.S., Poon R.W., Wong B.H., Tsoi H.W., Yip B.C., Huang Y., Chan K.H., Yuen K.Y. (2006). Molecular diversity of coronaviruses in bats. Virology.

[27] Huang C., Liu W.J., Xu W., Jin T., Zhao Y., Song J., Shi Y., Ji W., Jia H., Zhou Y. (2016). A Bat-Derived Putative Cross-Family Recombinant Coronavirus with a Reovirus Gene. PLoS Pathog..

[28] Woo P.C., Wang M., Lau S.K., Xu H., Poon R.W., Guo R., Wong B.H., Gao K., Tsoi H.W., Huang Y. (2007). Comparative analysis of twelve genomes of three novel group 2c and group 2d coronaviruses reveals unique group and subgroup features. J. Virol..

[29] Anthony S.J., Gilardi K., Menachery V.D., Goldstein T., Ssebide B., Mbabazi R., Navarrete-Macias I., Liang E., Wells H., Hicks A. (2017). Further Evidence for Bats as the Evolutionary Source of Middle East Respiratory Syndrome Coronavirus. MBio.

[30] Yang X.L., Hu B., Wang B., Wang M.N., Zhang Q., Zhang W., Wu L.J., Ge X.Y., Zhang Y.Z., Daszak P. (2015). Isolation and Characterization of a Novel Bat Coronavirus Closely Related to the Direct Progenitor of Severe Acute Respiratory Syndrome Coronavirus. J. Virol..

[31] Yang L., Wu Z., Ren X., Yang F., Zhang J., He G., Dong J., Sun L., Zhu Y., Zhang S. (2014). MERS-related betacoronavirus in Vespertilio superans bats, China. Emerg. Infect. Dis..

[32] Moreno A., Lelli D., De Sabato L., Zaccaria G., Boni A., Sozzi E., Prosperi A., Lavazza A., Cella E., Castrucci M.R. (2018). Detection and full genome characterization of two beta CoV viruses related to Middle East respiratory syndrome from bats in Italy (vol 14, 1, 2017). Virol. J..

[33] De Benedictis P., Marciano S., Scaravelli D., Priori P., Zecchin B., Capua I., Monne I., Cattoli G. (2014). Alpha and lineage C betaCoV infections in Italian bats. Virus Genes.

[34] Bourgarel M., Pfukenyi D.M., Boue V., Talignani L., Chiweshe N., Diop F., Caron A., Matope G., Misse D., Liegeois F. (2018). Circulation of Alphacoronavirus, Betacoronavirus and Paramyxovirus in Hipposideros bat species in Zimbabwe. Infect. Genet. Evol..

[35] Lelli D., Papetti A., Sabelli C., Rosti E., Moreno A., Boniotti M.B. (2013). Detection of coronaviruses in bats of various species in Italy. Viruses.

[36] Falcon A., Vazquez-Moron S., Casas I., Aznar C., Ruiz G., Pozo F., Perez-Brena P., Juste J., Ibanez C., Garin I. (2011). Detection of alpha and betacoronaviruses in multiple Iberian bat species. Arch. Virol..

[37] Tsuda S., Watanabe S., Masangkay J.S., Mizutani T., Alviola P., Ueda N., Iha K., Taniguchi S., Fujii H., Kato K. (2012). Genomic and serological detection of bat coronavirus from bats in the Philippines. Arch. Virol..

[38] Smith C.S., de Jong C.E., Meers J., Henning J., Wang L., Field H.E. (2016). Coronavirus Infection and Diversity in Bats in the Australasian Region. Ecohealth.

[39] Carrington C.V., Foster J.E., Zhu H.C., Zhang J.X., Smith G.J., Thompson N., Auguste A.J., Ramkissoon V., Adesiyun A.A., Guan Y. (2008). Detection and phylogenetic analysis of group 1 coronaviruses in South American bats. Emerg. Infect. Dis..

[40] Suzuki J., Sato R., Kobayashi T., Aoi T., Harasawa R. (2014). Group B betacoronavirus in rhinolophid bats, Japan. J. Vet. Med. Sci..

[41] Lacroix A., Duong V., Hul V., San S., Davun H., Omaliss K., Chea S., Hassanin A., Theppangna W., Silithammavong S. (2017). Genetic diversity of coronaviruses in bats in Lao PDR and Cambodia. Infect. Genet. Evol..

[42] Tao Y., Shi M., Chommanard C., Queen K., Zhang J., Markotter W., Kuzmin I.V., Holmes E.C., Tong S. (2017). Surveillance of Bat Coronaviruses in Kenya Identifies Relatives of Human Coronaviruses NL63 and 229E and Their Recombination History. J. Virol..

[43] Lau S.K., Woo P.C., Li K.S., Huang Y., Tsoi H.W., Wong B.H., Wong S.S., Leung S.Y., Chan K.H., Yuen K.Y. (2005). Severe acute respiratory syndrome coronavirus-like virus in Chinese horseshoe bats. Proc. Natl. Acad. Sci. USA.

[44] Rihtaric D., Hostnik P., Steyer A., Grom J., Toplak I. (2010). Identification of SARS-like coronaviruses in horseshoe bats (Rhinolophus hipposideros) in Slovenia. Arch. Virol..

[45] Li W., Shi Z., Yu M., Ren W., Smith C., Epstein J.H., Wang H., Crameri G., Hu Z., Zhang H. (2005). Bats are natural reservoirs of SARS-like coronaviruses. Science.

[46] Woo P.C., Lau S.K., Li K.S., Tsang A.K., Yuen K.Y. (2012). Genetic relatedness of the novel human group C betacoronavirus to Tylonycteris bat coronavirus HKU4 and Pipistrellus bat coronavirus HKU5. Emerg. Microbes. Infect..

[47] Lau S.K., Li K.S., Tsang A.K., Lam C.S., Ahmed S., Chen H., Chan K.H., Woo P.C., Yuen K.Y. (2013). Genetic Characterization of Betacoronavirus Lineage C Viruses in Bats Reveals Marked Sequence Divergence in the Spike Protein of Pipistrellus Bat Coronavirus HKU5 in Japanese Pipistrelle: Implications for the Origin of the Novel Middle East Respiratory Syndrome Coronavirus. J. Virol..

[48] Cui J., Li F., Shi Z.L. (2018). Origin and evolution of pathogenic coronaviruses. Nat. Rev. Microbiol..

[49] Song Z., Xu Y., Bao L., Zhang L., Yu P., Qu Y., Zhu H., Zhao W., Han Y., Qin C. (2019). From SARS to MERS, Thrusting Coronaviruses into the Spotlight. Viruses.

[50] Banerjee A., Kulcsar K., Misra V., Frieman M., Mossman K. (2019). Bats and Coronaviruses. Viruses.

[51] Woo P.C., Lau S.K., Lam C.S., Lau C.C., Tsang A.K., Lau J.H., Bai R., Teng J.L., Tsang C.C., Wang M. (2012). Discovery of seven novel Mammalian and avian coronaviruses in the genus deltacoronavirus supports bat coronaviruses as the gene source of alphacoronavirus and betacoronavirus and avian coronaviruses as the gene source of gammacoronavirus and deltacoronavirus. J. Virol..

[52] Wu Z., Yang L., Ren X., He G., Zhang J., Yang J., Qian Z., Dong J., Sun L., Zhu Y. (2016). Deciphering the bat virome catalog to better understand the ecological diversity of bat viruses and the bat origin of emerging infectious diseases. ISME J..

[53] Lau S.K.P., Woo P.C.Y., Li K.S.M., Tsang A.K.L., Fan R.Y.Y., Luk H.K.H., Cai J.-P., Chan K.-H., Zheng B.-J., Wang M. (2014). Discovery of a Novel Coronavirus, China Rattus Coronavirus HKU24, from Norway Rats Supports the Murine Origin of Betacoronavirus 1 and Has Implications for the Ancestor of Betacoronavirus Lineage A. J. Virol..

[54] Zhao G.P. (2007). SARS molecular epidemiology: A Chinese fairy tale of controlling an emerging zoonotic disease in the genomics era. Philos. Trans. R. Soc. Lond. B Biol. Sci..

[55] Wang M., Yan M., Xu H., Liang W., Kan B., Zheng B., Chen H., Zheng H., Xu Y., Zhang E. (2005). SARS-CoV infection in a restaurant from palm civet. Emerg. Infect. Dis..

[56] Song H.D., Tu C.C., Zhang G.W., Wang S.Y., Zheng K., Lei L.C., Chen Q.X., Gao Y.W., Zhou H.Q., Xiang H. (2005). Cross-host evolution of severe acute respiratory syndrome coronavirus in palm civet and human. Proc. Natl. Acad. Sci. USA.

[57] Guan Y., Zheng B.J., He Y.Q., Liu X.L., Zhuang Z.X., Cheung C.L., Luo S.W., Li P.H., Zhang L.J., Guan Y.J. (2003). Isolation and characterization of viruses related to the SARS coronavirus from animals in southern China. Science.

[58] Lima S.L., O’Keefe J.M. (2013). Do predators influence the behaviour of bats?. Biol. Rev..

[59] Li W., Wong S.K., Li F., Kuhn J.H., Huang I.C., Choe H., Farzan M. (2006). Animal origins of the severe acute respiratory syndrome coronavirus: Insight from ACE2-S-protein interactions. J. Virol..

[60] Luo Y., Li B., Jiang R.D., Hu B.J., Luo D.S., Zhu G.J., Hu B., Liu H.Z., Zhang Y.Z., Yang X.L. (2018). Longitudinal Surveillance of Betacoronaviruses in Fruit Bats in Yunnan Province, China During 2009-2016. Virol. Sin..

[61] Luo C.M., Wang N., Yang X.L., Liu H.Z., Zhang W., Li B., Hu B., Peng C., Geng Q.B., Zhu G.J. (2018). Discovery of Novel Bat Coronaviruses in South China That Use the Same Receptor as Middle East Respiratory Syndrome Coronavirus. J. Virol..

[62] Wang L., Fu S., Cao Y., Zhang H., Feng Y., Yang W., Nie K., Ma X., Liang G. (2017). Discovery and genetic analysis of novel coronaviruses in least horseshoe bats in southwestern China. Emerg. Microbes. Infect..

[63] Pan Y., Tian X., Qin P., Wang B., Zhao P., Yang Y.L., Wang L., Wang D., Song Y., Zhang X. (2017). Discovery of a novel swine enteric alphacoronavirus (SeACoV) in southern China. Vet. Microbiol..

[64] Hu B., Zeng L.P., Yang X.L., Ge X.Y., Zhang W., Li B., Xie J.Z., Shen X.R., Zhang Y.Z., Wang N. (2017). Discovery of a rich gene pool of bat SARS-related coronaviruses provides new insights into the origin of SARS coronavirus. PLoS Pathog..

[65] Lau S.K., Poon R.W., Wong B.H., Wang M., Huang Y., Xu H., Guo R., Li K.S., Gao K., Chan K.H. (2010). Coexistence of different genotypes in the same bat and serological characterization of Rousettus bat coronavirus HKU9 belonging to a novel Betacoronavirus subgroup. J. Virol..

[66] Widagdo W., Begeman L., Schipper D., Run P.R.V., Cunningham A.A., Kley N., Reusken C.B., Haagmans B.L., van den Brand J.M.A. (2017). Tissue Distribution of the MERS-Coronavirus Receptor in Bats. Sci. Rep..

[67] Dhondt K.P., Horvat B. (2013). Henipavirus infections: Lessons from animal models. Pathogens.

[68] Subudhi S., Rapin N., Bollinger T.K., Hill J.E., Donaldson M.E., Davy C.M., Warnecke L., Turner J.M., Kyle C.J., Willis C.K.R. (2017). A persistently infecting coronavirus in hibernating Myotis lucifugus, the North American little brown bat. J. Gen. Virol..

[69] Watanabe S., Masangkay J.S., Nagata N., Morikawa S., Mizutani T., Fukushi S., Alviola P., Omatsu T., Ueda N., Iha K. (2010). Bat coronaviruses and experimental infection of bats, the Philippines. Emerg. Infect. Dis..

[70] Sabir J.S., Lam T.T., Ahmed M.M., Li L., Shen Y., Abo-Aba S.E., Qureshi M.I., Abu-Zeid M., Zhang Y., Khiyami M.A. (2016). Co-circulation of three camel coronavirus species and recombination of MERS-CoVs in Saudi Arabia. Science.

[71] Plowright R.K., Eby P., Hudson P.J., Smith I.L., Westcott D., Bryden W.L., Middleton D., Reid P.A., McFarlane R.A., Martin G. (2015). Ecological dynamics of emerging bat virus spillover. Proc. Biol. Sci..

[72] Geller C., Varbanov M., Duval R.E. (2012). Human coronaviruses: Insights into environmental resistance and its influence on the development of new antiseptic strategies. Viruses.

[73] Sinclair R., Boone S.A., Greenberg D., Keim P., Gerba C.P. (2008). Persistence of category A select agents in the environment. Appl. Environ. Microbiol..

[74] Ge X.Y., Li J.L., Yang X.L., Chmura A.A., Zhu G., Epstein J.H., Mazet J.K., Hu B., Zhang W., Peng C. (2013). Isolation and characterization of a bat SARS-like coronavirus that uses the ACE2 receptor. Nature.

[75] Li W., Zhang C., Sui J., Kuhn J.H., Moore M.J., Luo S., Wong S.K., Huang I.C., Xu K., Vasilieva N. (2005). Receptor and viral determinants of SARS-coronavirus adaptation to human ACE2. EMBO J..

[76] Kuba K., Imai Y., Rao S., Gao H., Guo F., Guan B., Huan Y., Yang P., Zhang Y., Deng W. (2005). A crucial role of angiotensin converting enzyme 2 (ACE2) in SARS coronavirus-induced lung injury. Nat. Med..

[77] Yuan Y., Cao D.F., Zhang Y.F., Ma J., Qi J.X., Wang Q.H., Lu G.W., Wu Y., Yan J.H., Shi Y. (2017). Cryo-EM structures of MERS-CoV and SARS-CoV spike glycoproteins reveal the dynamic receptor binding domains. Nat. Commun..

[78] Raj V.S., Mou H., Smits S.L., Dekkers D.H., Muller M.A., Dijkman R., Muth D., Demmers J.A., Zaki A., Fouchier R.A. (2013). Dipeptidyl peptidase 4 is a functional receptor for the emerging human coronavirus-EMC. Nature.

[79] Lu G.W., Hu Y.W., Wang Q.H., Qi J.X., Gao F., Li Y., Zhang Y.F., Zhang W., Yuan Y., Bao J.K. (2013). Molecular basis of binding between novel human coronavirus MERS-CoV and its receptor CD26. Nature.

[80] Wang N., Shi X., Jiang L., Zhang S., Wang D., Tong P., Guo D., Fu L., Cui Y., Liu X. (2013). Structure of MERS-CoV spike receptor-binding domain complexed with human receptor DPP4. Cell Res..

[81] Lin H.X., Feng Y., Wong G., Wang L., Li B., Zhao X., Li Y., Smaill F., Zhang C. (2008). Identification of residues in the receptor-binding domain (RBD) of the spike protein of human coronavirus NL63 that are critical for the RBD-ACE2 receptor interaction. J. Gen. Virol..

[82] Li W., Sui J., Huang I.C., Kuhn J.H., Radoshitzky S.R., Marasco W.A., Choe H., Farzan M. (2007). The S proteins of human coronavirus NL63 and severe acute respiratory syndrome coronavirus bind overlapping regions of ACE2. Virology.

[83] Smith M.K., Tusell S., Travanty E.A., Berkhout B., van der Hoek L., Holmes K.V. (2006). Human angiotensin-converting enzyme 2 (ACE2) is a receptor for human respiratory coronavirus NL63. Adv. Exp. Med. Biol..

[84] Pohlmann S., Gramberg T., Wegele A., Pyrc K., van der Hoek L., Berkhout B., Hofmann H. (2006). Interaction between the spike protein of human coronavirus NL63 and its cellular receptor ACE2. Adv. Exp. Med. Biol..

[85] Lachance C., Arbour N., Cashman N.R., Talbot P.J. (1998). Involvement of aminopeptidase N (CD13) in infection of human neural cells by human coronavirus 229E. J. Virol..

[86] Kolb A.F., Hegyi A., Siddell S.G. (1997). Identification of residues critical for the human coronavirus 229E receptor function of human aminopeptidase N. J. Gen. Virol..

[87] Yeager C.L., Ashmun R.A., Williams R.K., Cardellichio C.B., Shapiro L.H., Look A.T., Holmes K.V. (1992). Human aminopeptidase N is a receptor for human coronavirus 229E. Nature.

[88] Delmas B., Gelfi J., L’Haridon R., Vogel L.K., Sjostrom H., Noren O., Laude H. (1992). Aminopeptidase N is a major receptor for the entero-pathogenic coronavirus TGEV. Nature.

[89] Williams R.K., Jiang G.S., Holmes K.V. (1991). Receptor for mouse hepatitis virus is a member of the carcinoembryonic antigen family of glycoproteins. Proc. Natl. Acad. Sci. USA.

[90] Dveksler G.S., Pensiero M.N., Cardellichio C.B., Williams R.K., Jiang G.S., Holmes K.V., Dieffenbach C.W. (1991). Cloning of the mouse hepatitis virus (MHV) receptor: Expression in human and hamster cell lines confers susceptibility to MHV. J. Virol..

[91] Peng G.Q., Xu L.Q., Lin Y.L., Chen L., Pasquarella J.R., Holmes K.V., Li F. (2012). Crystal Structure of Bovine Coronavirus Spike Protein Lectin Domain. J. Biol. Chem..

[92] Schultze B., Gross H.J., Brossmer R., Herrler G. (1991). The S-Protein of Bovine Coronavirus Is a Hemagglutinin Recognizing 9-O-Acetylated Sialic-Acid as a Receptor Determinant. J. Virol..

[93] Yang Y., Du L., Liu C., Wang L., Ma C., Tang J., Baric R.S., Jiang S., Li F. (2014). Receptor usage and cell entry of bat coronavirus HKU4 provide insight into bat-to-human transmission of MERS coronavirus. Proc. Natl. Acad. Sci. USA.

[94] Wang Q., Qi J., Yuan Y., Xuan Y., Han P., Wan Y., Ji W., Li Y., Wu Y., Wang J. (2014). Bat origins of MERS-CoV supported by bat coronavirus HKU4 usage of human receptor CD26. Cell Host Microbe..

[95] Menachery V.D., Yount B.L., Sims A.C., Debbink K., Agnihothram S.S., Gralinski L.E., Graham R.L., Scobey T., Plante J.A., Royal S.R. (2016). SARS-like WIV1-CoV poised for human emergence. Proc. Natl. Acad. Sci. USA.

[96] Barlan A., Zhao J., Sarkar M.K., Li K., McCray P.B., Perlman S., Gallagher T. (2014). Receptor variation and susceptibility to Middle East respiratory syndrome coronavirus infection. J. Virol..

[97] Tusell S.M., Schittone S.A., Holmes K.V. (2007). Mutational analysis of aminopeptidase N, a receptor for several group 1 coronaviruses, identifies key determinants of viral host range. J. Virol..

[98] Lau S.K.P., Fan R.Y.Y., Luk H.K.H., Zhu L., Fung J., Li K.S.M., Wong E.Y.M., Ahmed S.S., Chan J.F.W., Kok R.K.H. (2018). Replication of MERS and SARS coronaviruses in bat cells offers insights to their ancestral origins. Emerg. Microbes. Infect..

[99] van Doremalen N., Miazgowicz K.L., Milne-Price S., Bushmaker T., Robertson S., Scott D., Kinne J., McLellan J.S., Zhu J., Munster V.J. (2014). Host species restriction of Middle East respiratory syndrome coronavirus through its receptor, dipeptidyl peptidase 4. J. Virol..

[100] Zhou P., Fan H., Lan T., Yang X.L., Shi W.F., Zhang W., Zhu Y., Zhang Y.W., Xie Q.M., Mani S. (2018). Fatal swine acute diarrhoea syndrome caused by an HKU2-related coronavirus of bat origin. Nature.

[101] Gong L., Li J., Zhou Q., Xu Z., Chen L., Zhang Y., Xue C., Wen Z., Cao Y. (2017). A New Bat-HKU2-like Coronavirus in Swine, China, 2017. Emerg. Infect. Dis..

[102] Tu C., Crameri G., Kong X., Chen J., Sun Y., Yu M., Xiang H., Xia X., Liu S., Ren T. (2004). Antibodies to SARS coronavirus in civets. Emerg. Infect. Dis..

[103] Jeong J., Smith C.S., Peel A.J., Plowright R.K., Kerlin D.H., McBroom J., McCallum H. (2017). Persistent infections support maintenance of a coronavirus in a population of Australian bats (Myotis macropus). Epidemiol. Infect..

[104] Hall R.J., Wang J., Peacey M., Moore N.E., McInnes K., Tompkins D.M. (2014). New alphacoronavirus in Mystacina tuberculata bats, New Zealand. Emerg. Infect. Dis..

[105] Anthony S.J., Ojeda-Flores R., Rico-Chavez O., Navarrete-Macias I., Zambrana-Torrelio C.M., Rostal M.K., Epstein J.H., Tipps T., Liang E., Sanchez-Leon M. (2013). Coronaviruses in bats from Mexico. J. Gen. Virol..

[106] Fischer K., Zeus V., Kwasnitschka L., Kerth G., Haase M., Groschup M.H., Balkema-Buschmann A. (2016). Insectivorous bats carry host specific astroviruses and coronaviruses across different regions in Germany. Infect Genet. Evol..

[107] Monchatre-Leroy E., Boue F., Boucher J.M., Renault C., Moutou F., Ar Gouilh M., Umhang G. (2017). Identification of Alpha and Beta Coronavirus in Wildlife Species in France: Bats, Rodents, Rabbits, and Hedgehogs. Viruses.

[108] Misra V., Dumonceaux T., Dubois J., Willis C., Nadin-Davis S., Severini A., Wandeler A., Lindsay R., Artsob H. (2009). Detection of polyoma and corona viruses in bats of Canada. J. Gen. Virol..

[109] Wacharapluesadee S., Duengkae P., Rodpan A., Kaewpom T., Maneeorn P., Kanchanasaka B., Yingsakmongkon S., Sittidetboripat N., Chareesaen C., Khlangsap N. (2015). Diversity of coronavirus in bats from Eastern Thailand. Virol. J..

[110] Wacharapluesadee S., Sintunawa C., Kaewpom T., Khongnomnan K., Olival K.J., Epstein J.H., Rodpan A., Sangsri P., Intarut N., Chindamporn A. (2013). Group C betacoronavirus in bat guano fertilizer, Thailand. Emerg. Infect. Dis..

[111] Ksiazek T.G., Erdman D., Goldsmith C.S., Zaki S.R., Peret T., Emery S., Tong S., Urbani C., Comer J.A., Lim W. (2003). A Novel Coronavirus Associated with Severe Acute Respiratory Syndrome. N. Engl. J. Med..

[112] de Groot R.J., Baker S.C., Baric R.S., Brown C.S., Drosten C., Enjuanes L., Fouchier R.A., Galiano M., Gorbalenya A.E., Memish Z.A. (2013). Middle East respiratory syndrome coronavirus (MERS-CoV): Announcement of the Coronavirus Study Group. J. Virol..

[113] Lau S.K.P., Wong A.C.P., Lau T.C.K., Woo P.C.Y. (2017). Molecular Evolution of MERS Coronavirus: Dromedaries as a Recent Intermediate Host or Long-Time Animal Reservoir?. Int. J. Mol. Sci..

[114] Bates P., Bumrungsri S., Csorba G. (2008). *Rhinolophus* *thomasi*. The IUCN Red List of Threatened Species.

[115] Smith A.T., Johnston C.H., Jones G., Rossiter S. (2008). *Rhinolophus* *rex*. The IUCN Red List of Threatened Species.

[116] Hutson A.M., Kingston T., Walston J. (2008). *Rhinolophus* *pusillus*. The IUCN Red List of Threatened Species.

[117] Bates P., Bumrungsri S., Csorba G. (2008). *Rhinolophus* *pearsonii*. The IUCN Red List of Threatened Species.

[118] Alcaldé J., Benda P., Juste J. (2016). *Rhinolophus* *mehelyi*. The IUCN Red List of Threatened Species.

[119] Molur S., Srinivasulu C., Francis C. (2008). *Rhinolophus* *macrotis*. The IUCN Red List of Threatened Species.

[120] Taylor P. (2016). *Rhinolophus* *hipposideros*. The IUCN Red List of Threatened Species.

[121] Monadjem A., Jacobs D. (2017). *Rhinolophus* *hildebrandtii*. The IUCN Red List of Threatened Species.

[122] Piraccini R. (2016). *Rhinolophus* *ferrumequinum*. The IUCN Red List of Threatened Species.

[123] Juste J., Alcaldé J. (2016). *Rhinolophus* *euryale*. The IUCN Red List of Threatened Species.

[124] Taylor P. (2016). *Rhinolophus* *blasii*. The IUCN Red List of Threatened Species.

[125] Walston J., Kingston T., Hutson A.M. (2008). *Rhinolophus* *affinis*. The IUCN Red List of Threatened Species.

[126] Ar Gouilh M., Puechmaille S.J., Diancourt L., Vandenbogaert M., Serra-Cobo J., Lopez Roig M., Brown P., Moutou F., Caro V., Vabret A. (2018). SARS-CoV related Betacoronavirus and diverse Alphacoronavirus members found in western old-world. Virology.

[127] Pauly M., Pir J.B., Loesch C., Sausy A., Snoeck C.J., Hubschen J.M., Muller C.P. (2017). Novel Alphacoronaviruses and Paramyxoviruses Cocirculate with Type 1 and Severe Acute Respiratory System (SARS)-Related Betacoronaviruses in Synanthropic Bats of Luxembourg. Appl. Environ. Microbiol..

[128] Tang X.C., Zhang J.X., Zhang S.Y., Wang P., Fan X.H., Li L.F., Li G., Dong B.Q., Liu W., Cheung C.L. (2006). Prevalence and genetic diversity of coronaviruses in bats from China. J. Virol..

[129] Drexler J.F., Gloza-Rausch F., Glende J., Corman V.M., Muth D., Goettsche M., Seebens A., Niedrig M., Pfefferle S., Yordanov S. (2010). Genomic Characterization of Severe Acute Respiratory Syndrome-Related Coronavirus in European Bats and Classification of Coronaviruses Based on Partial RNA-Dependent RNA Polymerase Gene Sequences. J. Virol..

[130] Quan P.L., Firth C., Street C., Henriquez J.A., Petrosov A., Tashmukhamedova A., Hutchison S.K., Egholm M., Osinubi M.O., Niezgoda M. (2010). Identification of a severe acute respiratory syndrome coronavirus-like virus in a leaf-nosed bat in Nigeria. MBio.

[131] Lee S., Jo S.D., Son K., An I., Jeong J., Wang S.J., Kim Y., Jheong W., Oem J.K. (2018). Genetic Characteristics of Coronaviruses from Korean Bats in 2016. Microb. Ecol..

[132] Mendenhall I.H., Borthwick S., Neves E.S., Low D., Linster M., Liang B., Skiles M., Jayakumar J., Han H., Gunalan V. (2017). Identification of a Lineage D Betacoronavirus in Cave Nectar Bats (Eonycteris spelaea) in Singapore and an Overview of Lineage D Reservoir Ecology in SE Asian Bats. Transbound Emerg. Dis..

[133] Chen Y.N., Phuong V.N., Chen H.C., Chou C.H., Cheng H.C., Wu C.H. (2016). Detection of the Severe Acute Respiratory Syndrome-Related Coronavirus and Alphacoronavirus in the Bat Population of Taiwan. Zoonoses Public Health.

[134] Yang L., Wu Z., Ren X., Yang F., He G., Zhang J., Dong J., Sun L., Zhu Y., Du J. (2013). Novel SARS-like betacoronaviruses in bats, China, 2011. Emerg. Infect. Dis..

[135] Lau S.K., Feng Y., Chen H., Luk H.K., Yang W.H., Li K.S., Zhang Y.Z., Huang Y., Song Z.Z., Chow W.N. (2015). Severe Acute Respiratory Syndrome (SARS) Coronavirus ORF8 Protein Is Acquired from SARS-Related Coronavirus from Greater Horseshoe Bats through Recombination. J. Virol..

[136] Wu Z., Yang L., Ren X., Zhang J., Yang F., Zhang S., Jin Q. (2016). ORF8-Related Genetic Evidence for Chinese Horseshoe Bats as the Source of Human Severe Acute Respiratory Syndrome Coronavirus. J. Infect. Dis..

[137] Heinonen J., Vainio-Mattila K. (1997). Biodiversity/Ecotourism Assessments in Yunnan, China. Spec. Rep..

[138] Shi Z., Hu Z. (2008). A review of studies on animal reservoirs of the SARS coronavirus. Virus Res..

[139] Gonzalez J.P., Pourrut X., Leroy E. (2007). Ebolavirus and other filoviruses. Curr. Top. Microbiol. Immunol..

[140] Towner J.S., Amman B.R., Sealy T.K., Carroll S.A., Comer J.A., Kemp A., Swanepoel R., Paddock C.D., Balinandi S., Khristova M.L. (2009). Isolation of genetically diverse Marburg viruses from Egyptian fruit bats. PLoS Pathog..

[141] Smith C., Skelly C., Kung N., Roberts B., Field H. (2014). Flying-fox species density—A spatial risk factor for Hendra virus infection in horses in eastern Australia. PLoS ONE.

[142] Yob J.M., Field H., Rashdi A.M., Morrissy C., van der Heide B., Rota P., bin Adzhar A., White J., Daniels P., Jamaluddin A. (2001). Nipah virus infection in bats (order Chiroptera) in peninsular Malaysia. Emerg. Infect. Dis..

[143] Reynes J.M., Counor D., Ong S., Faure C., Seng V., Molia S., Walston J., Georges-Courbot M.C., Deubel V., Sarthou J.L. (2005). Nipah virus in Lyle’s flying foxes, Cambodia. Emerg. Infect. Dis..

[144] Chua K.B., Crameri G., Hyatt A., Yu M., Tompang M.R., Rosli J., McEachern J., Crameri S., Kumarasamy V., Eaton B.T. (2007). A previously unknown reovirus of bat origin is associated with an acute respiratory disease in humans. Proc. Natl. Acad. Sci. USA.

